# Dosimetric parameters of three new solid core I‐125 brachytherapy sources

**DOI:** 10.1120/jacmp.v3i2.2576

**Published:** 2002-03-01

**Authors:** Timothy D. Solberg, John J. DeMarco, Geoffrey Hugo, Robert E. Wallace

**Affiliations:** ^1^ Department of Radiation Oncology UCLA School of Medicine Los Angeles California 98095‐6951; ^2^ Jonsson Comprehensive Cancer Center UCLA School of Medicine Los Angeles California 90095‐6951

**Keywords:** Brachytherapy, I‐125, Monte Carlo, MCNP, Radiation Dosimetry

## Abstract

Monte Carlo calculations and TLD measurements have been performed for the purpose of characterizing dosimetric properties of new commercially available brachytherapy sources. All sources tested consisted of a solid core, upon which a thin layer of I125 has been adsorbed, encased within a titanium housing. The PharmaSeed BT‐125 source manufactured by Syncor is available in silver or palladium core configurations while the ADVANTAGE source from IsoAid has silver only. Dosimetric properties, including the dose rate constant, radial dose function, and anisotropy characteristics were determined according to the TG‐43 protocol. Additionally, the geometry function was calculated exactly using Monte Carlo and compared with both the point and line source approximations. The 1999 NIST standard was followed in determining air kerma strength. Dose rate constants were calculated to be 0.955±0.005,0.967±0.005, and $0.962\pm 0.005\;\text{cGy}h^{-1}U^{-1}$ for the PharmaSeed BT‐125‐1, BT‐125‐2, and ADVANTAGE sources, respectively. TLD measurements were in excellent agreement with Monte Carlo calculations. Radial dose function, *g*(*r*), calculated to a distance of 10 cm, and anisotropy function *F*(*r*, θ), calculated for radii from 0.5 to 7.0 cm, were similar among all source configurations. Anisotropy constants, ${\bar{\phi }}_{\text{an}}$, were calculated to be 0.941, 0.944, and 0.960 for the three sources, respectively. All dosimetric parameters were found to be in close agreement with previously published data for similar source configurations. The MCNP Monte Carlo code appears to be ideally suited to low energy dosimetry applications.

PACS number(s): 87.53.–j

## INTRODUCTION

Permanent implantation of I125 brachytherapy seeds has become an accepted and well‐documented method for the treatment of prostate cancer.^1^ ^–^ ^3^ Due to the low energy of the emitted radiation, obtaining the dosimetric data necessary for clinical implementation can be challenging. The American Association of Physicists in Medicine (AAPM), through the Radiation Therapy Committee recommends that “… the dosimetric characteristics of each new product (i.e., commercial source) be evaluated by at least one, and preferably two, independent investigators other than the manufacturer.”^4^ It is further recommended that “… a Monte Carlo study by an independent investigator should be made…”^4^ and that the dosimetric data be presented in accordance with the recommendations of AAPM Task Group 43.^5^ In this work, we report on Monte Carlo calculations of three different low energy brachytherapy sources. Calculations for the radial dose and anisotropy functions were performed in water following the AAPM Task Group 43 standard. The dose rate constant was determined as the ratio of the dose rate at 1 cm in water to the air kerma strength scored at a distance of 50 cm from the center of the source. Additionally, the geometry function was determined using Monte Carlo methodology by scoring the particle flux in vacuum as a function of distance from the source.

Accurate measurement of the dose distribution about an I125 source in phantom is complicated by strict requirements for precise dosimeter and source geometry and by spectral variation with distance from the source. Following previous work^6^ ^–^ ^10^ and that of others,^5^ ^,^ ^11^ ^–^ ^19^ thermoluminescent dosimeters (TLDs) were used in a precisely machined, water equivalent, solid phantom to measure the dose distributions of the BT‐125‐1 and IsoAid ADVANTAGE sources; the BT‐125‐2 source is not yet in production. Reusable TLDs have advantages for this type of measurement: broad dose and dose‐rate latitude; small size allowing precise positioning in bores in a solid phantom; the small perturbations, due to their presence in phantom, of the radiation environment, and the measured dose have been characterized; and their relative response to C60o and I125 spectra is well established.^20^ The phantom material was selected for liquid water equivalency.^5^ ^,^ ^9^ ^,^ ^21^ ^–^ ^23^

## METHODS

### A. Source description

**Figure 1:**
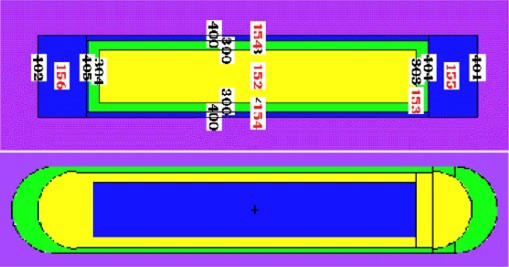
(Color) Construction of the Pharmaseed BT‐125 (top) and ADVANTAGE (bottom) I125 seeds are shown using the mcplot option in MCNP. The BT‐125 source was modeled with planar ends and the ADVANTAGE sourse with hemispherical ends, both per manufacturers' specifications. Numbers on the BT‐125 source designate the surfaces and volumes used in defining the MCNP geometry.

The geometry of each source was modeled per specifications provided by the manufacturers (Fig. 1). The ADVANTAGE source (IsoAid, Port Richey, Florida) consists of a cylindrical silver core, 0.30 cm long×0.05 cm in diameter, onto which a thin layer of I125 has been uniformly adsorbed. The silver core is sealed within a cylindrical titanium housing 0.45 cm in length×0.08 cm in diameter. The cylindrical portion of the titanium housing is 0.005 cm thick, with rounded titanium welds at each end.

Similarly, the Pharmaseed BT‐125‐1 (Syncor International Corporation, Woodland Hills, California) source consists of a cylindrical palladium core, 0.325 cm long×0.05 cm in diameter, onto which a 0.5 μm layer of I125 has been uniformly adsorbed. The palladium core is sealed within a cylindrical titanium housing 0.45 cm in length×0.08 cm in diameter. The cylindrical portion of the titanium housing is 0.006 cm thick, with 0.05 cm thick titanium welds, modeled as planar surfaces per manufacturer's specifications, at each end. Monte Carlo calculations on the BT‐125‐2 Syncor source, identical to the first with the exception of a solid Ag core, were also performed.

### B. Measurements

Two Syncor PharmaSeed model BT‐125‐1 I125 brachytherapy sources and one IsoAid Advantage 125I were used in performing TLD measurements. A NIST source calibration (1999 standard) was obtained for sources of each design manufactured in the same batch and transferred by one of two methods to the sources used in the measurements. For the Syncor sources, the secondary transfer was made through the agency of the manufacturer's calibration system. For the IsoAid source, the source used for the measurements was one of five having standard calibration at NIST, redistributed through the manufacturer to UCLA for this work. At the time of these measurements, the Syncor sources had air‐kerma strengths of $1.8U(1U=1)\text{cGy}-{\text{cm}}^{2}-h^{-1}$, or apparent activities of 1.42 mCi(1.27U=1.0 mCi), reported by the manufacturer with stated (NIST and manufacturer assay) uncertainty of 5%. The IsoAid source had an air‐kerma strength of 3.96 U (3.12 mCi) at the time of measurement, with an uncertainty of 0.5% (i.e., in the NIST WAFAC measurement of $S_{k}$).

In the course of this study, transverse axis dose distributions were measured in phantom at distances of 0.17 to 10.0 cm from the center of the source to determine the radial dose function. Dose at a distance of 1 cm transverse from the source center was measured to evaluate the dose‐rate constant. TLD calibration for the latter measurement was made with reference exposure in a (TG51) calibrated Cobalt60 standard beam (Theratronics International, Kanata, Ontario, Canada) and TLD relative energy response factors for the I125 spectrum were taken from prior work^6^ ^–^ ^9^ and ultimately from Weaver *et al*.^20^ In‐phantom exposures in the dose‐rate constant measurements were one to three days approximately in order to minimize uncertainty in source strength corrected for decay during exposure. That is, the effects of decay have been included in the analyses. The duration of in‐phantom exposure to assess the radial dose and anisotropy functions ranged from three to eighteen days. Measurements used TLD rods from a single, large, annealed batch in a water equivalent phantom for I125 or in Lucite build‐up capsules for C60o. The handling of TLDs, the design and water equivalence of the phantom, and the sources used are detailed elsewhere^9^ and summarized below. The geometry function, *G*(*r*, θ), was calculated for each brachytherapy source using the line‐source approximation of TG43.^5^

Lithium fluoride TLD‐100 rods (Harshaw‐Bicron, Solon, Ohio), 6 mm length×1 mm diameter, were used in all phantom measurements evaluating the model BT‐125‐1 and ADVANTAGE sources, and in Lucite build‐up capsules using the C60o source. An automated TLD reader was used (Harshaw‐Bicron Model QS‐5500). Corrections were made to rod response for finite dosimeter volume,^19^ ^,^ ^20^ energy response,^20^ and scaled for exposure duration79 Rods were placed in phantom in patterns to minimize inter‐rod effects.^7^ ^,^ ^19^

A phantom correction factor was applied to TLD responses in measurements of the dose‐rate constant, Λ. In the absolute measurement of the dose‐rate constant in the phantom, the correction factor was 0.995, calculated for the phantom material at 1 cm, and was applied to arrive at the dose‐rate constant in water.^9^ ^,^ ^23^ Correction factors that were applied to TLD response in the analysis of the radial dose function are found elsewhere.^23^

In any experiment, the TLD rods received approximately 50 to 500 cGy over one to eighteen days, estimated using the dose‐rate constants, Λ, for the sources found in this and prior work.^23^ Such doses are within the linear range stated by the manufacturer for the TLD‐100 rods.

For all measurements, a single batch of TLD rods was selected that demonstrated response uniformity in the absolute range of mean ±1.5–2%. No individual or batch calibrations were applied to the TLD responses when evaluating the radial dose function and the anisotropy function. Group (14 and 16 rods) calibration factors were applied for TLDs in the evaluation of the dose‐rate constant. Regarding TLD constancy, the Cobalt‐60 referenced calibration factors were within 3% of those for previous investigations.^7^ ^–^ ^9^ Prior to exposure, the full batch of TLD rods was annealed at 400 °C for 1 h, quenched to ambient room temperature in 30 mins, and heated at 80 °C for 24 h Annealing was repeated as necessary to complete all experiments.

**Figure 2:**
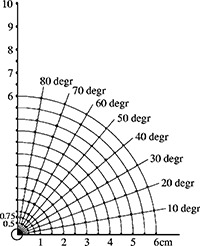
Schematic of TLD locations in measurement phantom. Each marked point represents a single 1.1 mm diam.×6.1 mm depth bore to accommodate TLD rods. The 5 mm diameter central seed carrier is depicted at the origin (see the text). Only a portion of the phantom is shown.

As in previous work,^9^ ^,^ ^10^ a phantom of Plastic Water PW2030 (Computerized Imaging Reference Systems, Norfolk, Virginia), was selected for dosimetric water equivalence for low‐energy sources, from 20 to 30 keV.^21^ ^–^ ^23^ This phantom material has been evaluated for water equivalence and correction factors derived.^9^ ^,^ ^23^ As in previous work,^9^ ^,^ ^10^ the water equivalent phantom, $30\times 30\times 7{\text{cm}}^{3}$, was designed with a central bore to accommodate one of two (PW2030 material) source carriers holding the source either parallel (to evaluate the dose‐rate constant) or perpendicular (to evaluate the dose distribution) to the long axes of the TLD rods. The phantom bores, depicted in Fig. 2, to hold TLD rods were arranged to sample a quadrant with respect to the source long axis, at angles from 0° to 90° (measured from the long axis of the source) in 10° steps and at 5 mm intervals from 5 to 100 mm, with an additional ring at 7.5 mm. Plugs of phantom material were used to fill the phantom gaps when TLD rods were absent in the measurements. The TLD bores also accommodated a brachytherapy source in the trials to assess the radial dose function.

For measurement of the dose‐rate constant, the source was held upright at the center of the concentric circular bore array in the phantom. Ten TLDs were placed in a ring at a radius of 1 cm from the source. Bore plugs filled the intervening phantom gaps.

Measurements to evaluate the radial dose function used a dosimeter placement pattern that was selected to obviate inter‐rod effects in phantom.^7^ ^–^ ^10^ In three trials using an offset source, the source was positioned in the bore at (r=1 cm,θ=80°) with 39 TLDs placed in line‐of‐sight of the source in selected phantom bores. In particular, dosimeters were placed in all positions (0.5 to 10 cm) along the ray in Fig. 2 at 90°, with additional dosimeters at (1.5, 2 cm; 80°), (0.75, 1, 1.5, 2, 2.5, 5; 70°), (1, 1.5, 2; 60°), (2.5; 40°), and (1; 20°). The arrangement places dosimeters 0.174 cm from the source at two locations per measurement, improving the numbers for data analysis. The same is true at distances of 0.292, 0.544, 1.0, 1.033, and 1.53 cm. Precision machining of the phantom and the bore array ensured geometric integrity of the measurement(s). In each type of measurement, the sources were positioned in phantom with no attention to orientation in prior measurements (i.e., the end pointing “up” or “out” was randomly selected).

Uncertainty in measured results for the dose‐rate constant, given as a standard deviation, can be expected to arise from each of the correction factors discussed above, statistical variation in the TLD responses, and from the given uncertainty in the provided air‐kerma strength, Sk. For the Syncor source, the uncertainty in $S_{k}$ is given as 5%. This value is 0.5% for the IsoAid source, owing to direct calibration at NIST. Statistical uncertainty in the TLD response(s) is 4–5%. Uncertainty in the energy correction factor (for TLD response) is 1–2%,^20^ phantom correction is 2%,^7^ ^–^ ^10^ ^,^ ^21^ ^–^ ^23^ volume correction is analytically taken to be exact at the TG43 reference point, (1 cm,π/2), as are inter‐chip corrections,^7^ ^–^ ^10^ ^,^ ^20^ and reference calibration of TLD in C60o is assigned an uncertainty of 0.5% (TG51 protocol). The combined uncertainty (from all factors) in the dose‐rate constant is therefore 4.8% for the IsoAid source and 7.7% for the Syncor source, where the uncertainty has been combined in quadrature. The inter‐chip correction and the phantom material correction vary with radial distance and were applied in the analysis of TLD responses that gives rise to the radial dose function providing a net uncertainty of 7–8% at the 95% confidence level. Given that the anisotropy function, *F*(*r*, θ) is defined to include the ratio of dose‐rates at a given radial distance, the inter‐chip and phantom material corrections cancel by division. The net uncertainty in the anisotropy data is then the statistical uncertainty in the TLD responses in those measurements, about 10%.

### C. Monte Carlo calculations

The Monte Carlo N‐Particle (MCNP) code, version 4C, was used to determine the relevant dosimetric parameters of the three source configurations. MCNP has its foundations as a neutron transport code beginning in the early days of the Manhattan Project.^24^ ^–^ ^26^ Later, the photon/electron transport in MCNP was based loosely on that of the Integrated Tiger Series (ITS version 1.0), which in turn has been adapted from ETRAN.^27^ With version 4B, MCNP features photon physics equivalent to that of the Integrated Tiger Series (ITS version 3.0). With the recently released version 4C, significant electron physics enhancements have been made to “make MCNP more current with the Integrated Tiger Series.”^28^ This includes improvements to radiative stopping powers and bremsstrahlung production. Though the Goudsmit‐Saunderson formalism for multiple electron scattering in ETRAN/ITS is considered superior to other approaches, some deficiencies, particularly with regard to energy‐loss straggling, have been noted.^29^ ^,^ ^30^ In the original implementation, energy‐loss straggling is inadequately sampled from the Blunck‐Leisegang approximation to the Landau theory. This deficiency was addressed in later versions of ITS^31^ and implemented in MCNP versions 4B and later.^28^ ^,^ ^32^

MCNP has a number of advantages that make it attractive for medical physics applications. The range for photon and electron transport extends from 1 keV to 100 MeV Important low energy phenomena, such as the production and transport of characteristic x‐rays and Auger cascades, are accurately modeled. The code also transports neutrons, with photoneutron production recently implemented in a separate release (MCNPX). MCNP supports several geometry schemes simultaneously; the combinatorial geometry which combines first and second degree surfaces and fourth degree elliptical tori is ideally suited to the modeling of complex brachytherapy sources. Additionally, a nested lattice feature mimics voxel‐based medical imagery. Fluence, kerma, or dose tallies can be simultaneously performed in simple cylindrical or spherical geometries or in a regular lattice geometry.

All directives from the user, including source configuration, target geometry, material specification, physics parameters, and biasing options, originate from a single text file; there is no user initiated written or compiled computer code. If necessary, modifications to the code can be easily performed through the aid of the pre‐processor. The ability to distribute a calculation over multiple, loosely connected computer processors allows the performance of MCNP to scale linearly with the number of processors dedicated to the task. MCNP is supported on numerous computer architectures and operating systems including Unix and Windows™ (DOS).

In this work, the detailed physics treatment that includes coherent scattering, form factors to account for electron binding effects, and generation of fluorescent photons after photoelectric absorption was used for all calculations. Fluorescent x‐rays following *K*‐ and *L*‐shell vacancies are generated and transported assuming an isotropic emission. Auger electrons are also generated and transported. The standard MCNP photoelectric cross sections were replaced with the more recent XCOM tabulation of Berger and Hubbell.^33^ ^,^ ^34^ For all calculations, electron transport was not explicitly considered, thus with electron transport turned off, a thick target bremsstrahlung model, in which electrons are immediately annihilated but assumed to travel in the direction of the incident photon, was used. Similarly, Auger electrons deposit their energy locally. For all calculations, the modified I125 decay spectrum was taken from that of Attix.^35^ The spectrum consisted of five energies in the following abundance: 22.1 keV (14%), 25.0 keV (4%), 27.4 keV (64%), 31.1 keV (14%), 35.5 keV (4%).

Per AAPM Task Group 43, the dose rate constant is defined as the ratio of the dose rate at a reference distance per unit air kerma strength:

(1) $$\Lambda =\dot{D}(r_{o},\theta_{o})/S_{K}.$$

For Monte Carlo calculations, the dose rate was determined in water at a distance of 1 cm in a cylindrical annulus 0.05 cm thick×0.05 cm deep. The MCNP *f4 tally, a track length fluence estimator, was used to score the energy fluence $\left(\text{MeV}/{\text{cm}}^{2}\right)$ in the cylindrical annulus. Energy fluence was converted to dose rate using mass‐energy absorption coefficients obtained from Seltzer.^36^ These are also available from the National Institute of Standards and Technology (NIST) web site. Air‐kerma strength was scored in a similar cylindrical geometry 0.2 cm thick×0.2 cm deep at a radial distance of 50 cm from the center of the source. The intervening medium between the source and air‐filled scoring annulus consisted of a vacuum. For dose rate, $4.0\times 10^{7}$ primary particles were followed resulting in statistical uncertainty of less than 0.25% while for air kerma, 4.0×107 primary particles were followed resulting in statistical uncertainty of less than 0.5% (statistical uncertainty is reported as 1σ). Particle cutoff energy was set to 5.0 keV following the 1999 NIST WAFAC standard.^37^ ^,^ ^38^

For TLD measurements, the geometry function was calculated using the AAPM Task Group 43 approximation for a line source [Eq. (2)]. For Monte Carlo calculations, MCNP was used to determine *G*(*r*, θ) exactly (within the statistical limitations of the methodology). To accomplish this, a geometry was defined in which cylindrical annuli 0.05 cm thick×0.05 cm deep were positioned at distances from 0.5 to 10.0 from the center of the source. Using the MCNP f4 tally, particle fluence $\left(1/{\text{cm}}^{2}\right)$ was scored in each annulus in a vacuum outside the source. $2.0\times 10^{7}$ primary particles were followed resulting in statistical uncertainties ranging from 0.15% to 0.67% at 0.5 and 10.0 cm, respectively. Data were subsequently normalized for volume and to unity at 1.0 cm,

(2) $$G(r,\theta )=\{\begin{array}{l} \frac{1}{r^{2}}\;\;\;\;\;\text{point}\;\text{source}\;\text{approximation}\\ \frac{\beta }{Lr\sin\theta }\;\;\;\;\;\text{line}\;\text{source}\;\text{approximation}. \end{array}$$

In a manner analogous to the geometry function, the radial dose function was determined by scoring the dose rate in water in cylindrical annuli 0.05 cm thick×0.05 cm deep positioned at distances ranging from 0.5 to 10.0 cm from the center of the source. As described above, the MCNP *f4 tally was used to score energy fluence $\left(\text{MeV}/{\text{cm}}^{2}\right)$ in each cylindrical annulus. Energy fluence was converted to dose rate using mass‐energy absorption coefficients obtained from Seltzer.^36^ $2.0\times 10^{7}$ primary particles were followed resulting in statistical uncertainty of 0.5% or less in all scoring volumes. Dose rate was normalized to unity at a distance of $r_{0}=1.0\;\text{cm}$, and the radial dose function calculated according to Eq. (3) using the Monte Carlo geometry function determined above,

(3) $$g(r)=\frac{\dot{D}(r,\theta_{o})G(r_{o},\theta_{o})}{\dot{D}(r_{o},\theta_{o})G(r,\theta_{o})}.$$

The anisotropy function [Eq. (4)] was determined by scoring the dose rate in water in spherical volumes located in 5° increments, over a 90° range, at distances of 0.5, 1.0, 2.0, 3.0, 5.0, and 7.0 from the center of the source. As described above, the MCNP *f4 tally was used to score energy flux in each spherical volume which was converted to dose rate using mass‐energy absorption coefficients obtained from Seltzer.^36^ Due to the small size of each scoring volume, it was necessary to follow $1.0\times 10^{9}$ primary particles in order to achieve statistically meaningful results. Statistical uncertainties ranged from 0.10% to 2.00% at 0.5 and 7.0 cm, respectively. At each radial distance, dose rate was normalized to unity at an angle of 90°,

(4) $$F(r,\theta )=\frac{\dot{D}(r,\theta )G(r,\theta_{0})}{\dot{D}(r,\theta_{o})G(r,\theta )}.$$

## RESULTS

Measured and calculated dose rate constants are shown in Table I. For the two Syncor sources, Monte Carlo calculations yielded values of 0.955±0.005 and $0.967\pm 0.005\;\text{cGy}h^{-1}U^{-1}$ for the Pd and Ag core configurations, respectively. TLD results (Pd core only), $0.95\pm 0.07\;\text{cGy}h^{-1}U^{-1}$, agreed with Monte Carlo calculations within the uncertainty of the respective methods. Both measured and calculated results compared favorably with the prior study by Popescu et al. of an identical source (BT‐125‐1).^39^

**Table I:** Dose rate constants determined in the present work compared with that of previous studies in which solid core, cylindrical sources were evaluated.

Reference	Technique of determination	Medium	Dose rate constant Λ (cGy h^‐1^ U^‐1^)
Present work BT‐125‐1 (Pd‐core)	Monte	Carlo Water	0.955±0.005
Present work BT‐125‐1 (Pd‐core)	TLD Measurements	Plastic Water™	0.95±0.07
Popescu *et al*. (Ref. 35) BT‐125‐1 (Pd‐core)	Monte Carlo	Water	0.950±0.06
Popescu *et al*. (Ref. 35) BT‐125‐1 (Pd‐core)	TLD Measurements	Solid Water™	0.900±0.03
Present work BT‐125‐2 (Ag‐core)	Monte Carlo	Water	0.967±0.005
Present Work Advantage	Monte Carlo	Water	0.962±0.005
Present Work Advantage	TLD Measurements	Plastic Water™	0.96±0.05
Kirov and Williamson STM1251 (Ref. 36)	Monte Carlo	Water	0.980
Williamson *et al*. Model 6711 (Ref. 37)	TLD Measurements	Plastic Water™	0.980

Similarly, Monte Carlo and TLD results for the ADVANTAGE source, 0.962±0.005 and $0.96\pm 0.05\;\text{cGy}h^{-1}U^{-1}$, respectively, compare favorably with one another. Table I shows dose rate constant values for two additional, commercially available, solid‐core 125I sources, model STM1251 (Source Tech) and model 6711 (Nycomed‐Amersham). Results from the present work are consistent with those obtained from similar source configurations.^38^ ^,^ ^40^

One‐dimensional geometry factors determined using the point and line source approximations and determined directly through Monte Carlo methods are summarized in Table II. While all three methods converge to similar results as r→∞, significant differences can be observed close to the source, suggesting a potentially more appropriate methodology for normalizing subsequent radial dose and anisotropy data. This opinion was expressed previously by Rivard.^41^

**Table II:** Geometry function for the two sources evaluated in the present work, calculated using the point and line‐source approximations and using the Monte Carlo flux tally in a vacuum.

Geometry function, G(r,θ=90)					
		BT‐125‐1/2		Advantage	
Radial distance (cm)	Point source approximation $\left(1/r^{2}\right)$	Linear source approximation, TG‐43	Calculated, Monte Carlo	Linear source approximation, TG‐43	Calculated, Monte Carlo
0.50	4.000	3.927	3.668	3.886	3.908
1.00	1.000	1.000	1.000	0.993	1.000
1.50	0.444	0.446	0.453	0.443	0.447
2.00	0.250	0.251	0.256	0.250	0.252
2.50	0.160	0.161	0.165	0.160	0.162
3.00	0.111	0.112	0.114	0.111	0.112
3.50	0.082	0.082	0.084	0.082	0.082
4.00	0.063	0.063	0.065	0.062	0.063
4.50	0.049	0.050	0.051	0.049	0.050
5.00	0.040	0.040	0.041	0.040	0.040
6.00	0.028	0.028	0.029	0.028	0.028
7.00	0.020	0.020	0.021	0.020	0.021
8.00	0.016	0.016	0.016	0.016	0.016
9.00	0.012	0.012	0.013	0.012	0.012
10.00	0.010	0.010	0.010	0.010	0.010

**Figure 3:**
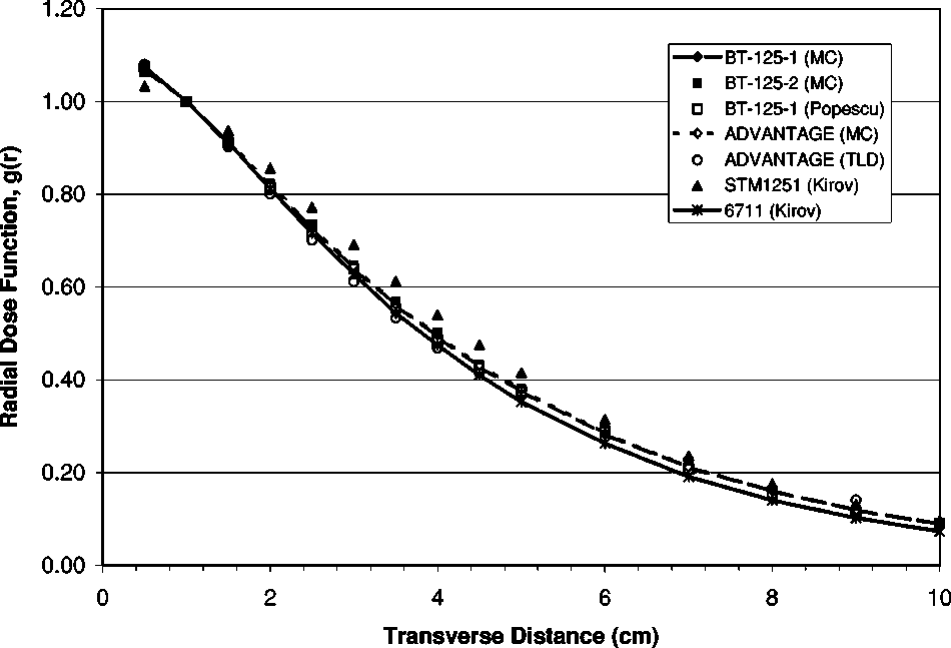
Radial dose function from the present work compared with that of previous studies in which solid core, cylindrical sources were evaluated. Monte Carlo data from the present work is designated MC, while measured data from the present work is designated TLD. Measured data at radii less than 0.5 cm are omitted for clarity.

The measured and calculated radial dose functions for the sources evaluated are shown graphically in Fig. 3. Excellent agreement is noted between the present work and results of several prior studies. In fact, it is interesting to note that the transverse dosimetric characteristics are nearly indistinguishable for all but the model STM1251 source which is slightly more penetrating. Data from the present work are summarized in Table III together with those reported in earlier studies on similar source configurations.

**Table III:** Radial dose functions determined in the present work compared with that of previous studies in which solid core, cylindrical sources were evaluated.

Radial dose function, g(r)								
		Present work				Popescu *et al*.	Kirov and Williamson	
Radial distance (cm)	BT‐125‐1 Monte Carlo	BT‐125‐1 Monte Carlo	BT‐125‐1 TLD	BT‐125‐2 Monte Carlo	Advantage Monte Carlo	Advantage TLD	STM1251 Monte Carlo	6711 Monte Carlo
0.5	1.069	1.066	1.062	1.074	1.080	1.073	1.033	1.074
1.0	1.000	1.000	1.000	1.000	1.000	1.000	1.000	1.000
1.5	0.908	0.913	0.913	0.914	0.902	0.910	0.937	0.912
2.0	0.816	0.817	0.823	0.822	0.800	0.814	0.856	0.812
2.5	0.727	0.720	0.735	0.729	0.701	0.721	0.772	0.716
3.0	0.640	0.630	0.647	0.645	0.611	0.639	0.691	0.628
3.5	0.561	0.549	0.568	0.563	0.533	0.554	0.612	0.544
4.0	0.493	0.479	0.501	0.496	0.468	0.483	0.540	0.475
4.5	0.428	0.420	0.433	0.431	0.414	0.425	0.475	0.410
5.0	0.375	0.372	0.380	0.379	0.368	0.374	0.415	0.352
6.0	0.282	0.300	0.285	0.284	0.294	0.289	0.314	0.263
7.0	0.211	0.247	0.213	0.212	0.227	0.207	0.236	0.191
8.0	0.160	0.207	0.160	0.160	0.165	0.152	0.176	0.140
9.0	0.119	0.197	0.120	0.119	0.141	0.115	0.131	0.102
10.0	0.089		0.088	0.088		0.090	0.096	0.073

**Figure 4:**
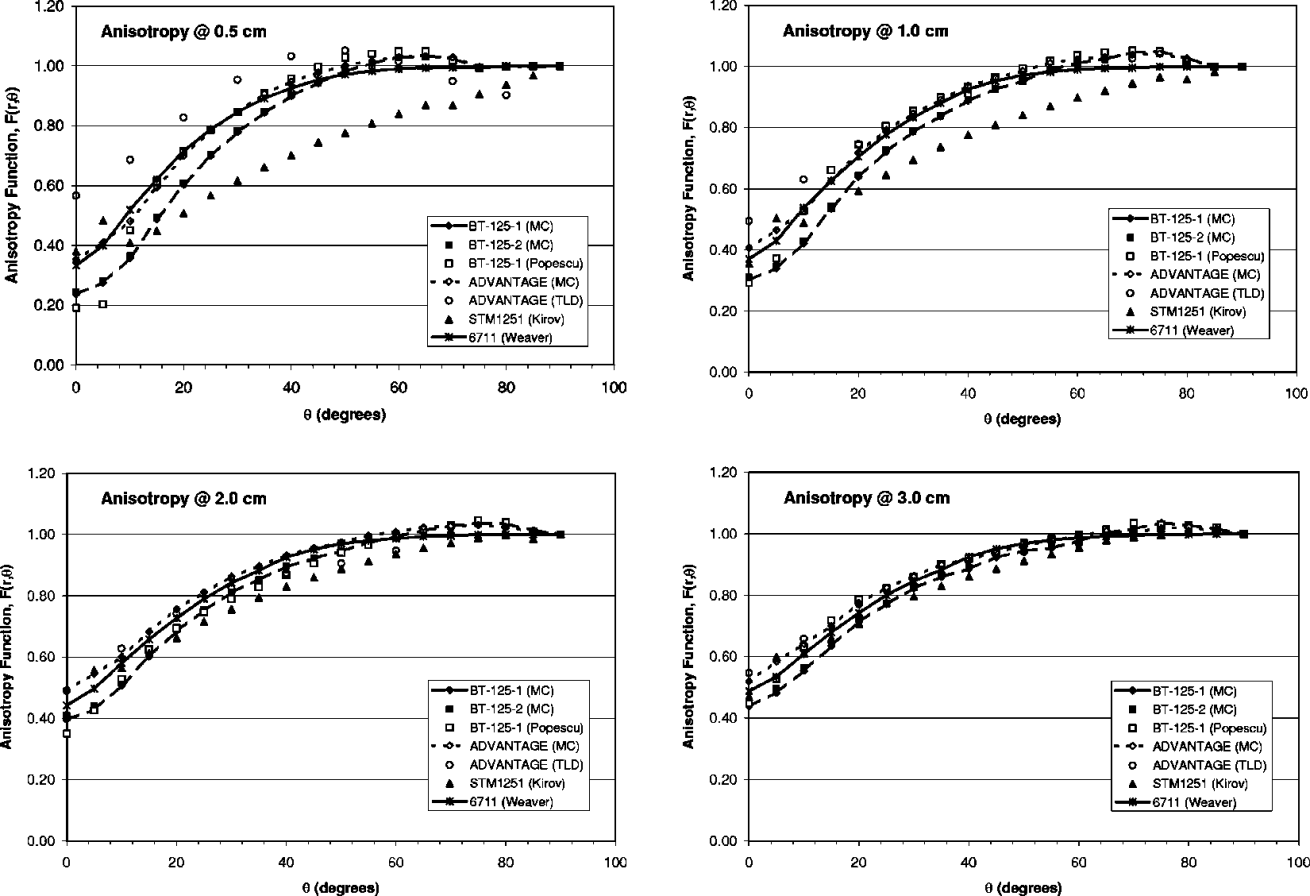
Anisotropy factors at radii of 0.0, 1.0, 2.0, and 3.0 cm with the present work compared to that of previous studies in which solid core, cylindrical sources were evaluated. Monte Carlo data from the present work is designated MC, while measured data from the present work is designated TLD.

Anisotropy data at radii of 0.5, 1.0, 2.0, and 3.0 cm are shown in Fig. 4. Data compare favorably with earlier studies, particularly those of Popescu *et al*. (BT‐125‐1) and Weaver (model 6711).^39^ ^,^ ^42^ At 0.5 cm radius, TLD results for the ADVANTAGE source reflect the difficulty in performing such measurements. Similarly, data suggest the model STM1251 source is somewhat more anisotropic than those evaluated in the present study, an observation pointed out earlier by Kirov.^40^

A complete set of anisotropy data determined by Monte Carlo calculations is tabulated in (Tables IV A, V A, and VI A. Additionally, at each radius Monte Carlo anisotropy data were fit to a fifth order polynomial. Fitting coefficients are listed in Tables IV B, V B, and VI B. Using the fitted data, the anisotropy factor, $\phi_{an}(r)$, was determined following Eq. (5). Anisotropy factors as a function of radius for several commercially available sources are listed in Table VII. Results from the present work compare favorably with those of previous studies evaluating similar or identical sources,^39^ ^,^ ^40^ ^,^ ^42^

**Table IV A:** Calculated anisotropy function for the BT‐125‐‐1 source determined by scoring the dose rate in water in spherical volumes located in 5° increments, over a 90° range, at distances of 0.5, 1.0, 2.0, 3.0, 5.0, and 7.0 from the center of the source.

Anisotropy function, F(r,θ) Distance from source center (cm)						
θ (deg)	0.5	1.0	2.0	3.0	5.0	7.0
0	0.237	0.301	0.396	0.438	0.501	0.557
5	0.275	0.339	0.428	0.483	0.557	0.592
10	0.358	0.421	0.507	0.554	0.617	0.638
15	0.487	0.535	0.602	0.635	0.681	0.689
20	0.603	0.639	0.685	0.715	0.730	0.743
25	0.699	0.721	0.752	0.771	0.811	0.788
30	0.779	0.786	0.811	0.823	0.825	0.824
35	0.844	0.838	0.853	0.860	0.851	0.881
40	0.900	0.888	0.895	0.886	0.896	0.875
45	0.943	0.926	0.922	0.923	0.921	0.901
50	0.983	0.952	0.946	0.945	0.942	0.928
55	1.009	0.989	0.973	0.953	0.942	0.932
60	1.030	1.011	0.993	0.977	0.957	0.959
65	1.035	1.025	1.013	1.004	0.993	1.000
70	1.026	1.046	1.027	1.016	1.003	0.985
75	0.993	1.049	1.037	1.035	1.031	1.026
80	1.000	1.028	1.035	1.028	1.011	1.034
85	0.999	1.001	1.014	1.016	1.006	1.015
90	1.000	1.000	1.000	1.000	1.000	1.000

**Table IV B:** Coefficients for anisotropy fnction fit to a fifth order polynomial.

Fitting coefficients for F(r, θ) Distance from source center (cm)						
	0.5	1.0	2.0	3.0	5.0	7.0
$a_{0}$	2.25E–25	2.89E–89	3.87E–87	4.33E–33	5.00E–00	5.57E–57
$a_{1}$	9.25E–25	9.72E–72	8.02E–02	9.22E–22	1.05E–05	4.25E–25
$a_{2}$	8.26E–26	7.38E–38	7.00E–00	5.39E–39	2.54E–54	5.57E–57
$a_{3}$	–2.36E–36	–2.47E–47	–2.45E–45	–2.09E–09	–1.23E–23	–2.00E–00
$a_{4}$	2.28E–28	2.90E–90	3.03E–03	2.70E–70	1.69E–69	2.62E–62
$a_{5}$	–7.64E–64	–1.22E–22	–1.32E–32	–1.21E–21	–7.81E–81	–1.19E–19

**Table V A:** Calculated anisotropy function for the BT‐125‐2 source determined by scoring the dose rate in water in spherical volumes located in 5° increments, over a 90° range, at distances of 0.5, 1.0, 2.0, 3.0, 5.0, and 7.0 from the center of the source.

Anisotropy function, F(r,θ) Distance from source center (cm)						
θ (deg)	0.5	1.0	2.0	3.0	5.0	7.0
0	0.244	0.310	0.408	0.452	0.532	0.557
5	0.282	0.347	0.441	0.498	0.573	0.590
10	0.365	0.428	0.515	0.563	0.633	0.662
15	0.494	0.542	0.610	0.644	0.692	0.703
20	0.609	0.644	0.690	0.721	0.744	0.778
25	0.704	0.726	0.756	0.772	0.833	0.811
30	0.783	0.788	0.810	0.824	0.840	0.869
35	0.848	0.838	0.852	0.864	0.873	0.864
40	0.902	0.890	0.897	0.890	0.917	0.942
45	0.944	0.926	0.924	0.928	0.933	0.927
50	0.983	0.951	0.947	0.943	0.957	0.965
55	1.009	0.987	0.971	0.959	0.963	0.959
60	1.027	1.010	0.991	0.983	0.969	0.956
65	1.031	1.023	1.014	1.006	1.004	1.004
70	1.012	1.042	1.025	1.017	1.019	1.026
75	0.993	1.043	1.033	1.026	1.018	1.023
80	0.999	1.018	1.030	1.028	1.012	1.011
85	1.000	1.001	1.005	1.017	1.014	1.030
90	1.000	1.000	1.000	1.000	1.000	1.000

**Table V B:** Coefficients for anisotropy function fit to a fifth order polynomial.

Fitting coefficients for F(r,θ) Distance from source center (cm)						
	0.5	1.0	2.0	3.0	5.0	7.0
$a_{0}$	2.32E–32	2.98E–98	3.99E–99	4.47E–47	5.29E–29	5.55E–55
$a_{1}$	9.02E–02	9.90E–90	8.33E–33	9.19E–19	7.84E–84	6.74E–74
$a_{2}$	8.49E–49	7.00E–00	6.26E–26	4.83E–83	4.04E–04	4.68E–68
$a_{3}$	–2.45E–45	–2.34E–34	–2.19E–19	–1.86E–86	–1.55E–55	–1.78E–78
$a_{4}$	2.39E–39	2.73E–73	2.68E–68	2.38E–38	1.95E–95	2.30E–30
$a_{5}$	–8.07E–07	–1.13E–13	–1.15E–15	–1.06E–06	–8.58E–58	– 1.03E–09

**Table VI A:** Calculated anisotropy function for the ADVANTAGE source determined by scoring the dose rate in water in spherical volumes located in 5° increments, over a 90° range, at distances of 0.5, 1.0, 2.0, 3.0, 5.0, and 7.0 from the center of the source.

Anisotropy function, F(r,θ) Distance from source center (cm)						
θ (deg)	0.5	1.0	2.0	3.0	5.0	7.0
0	0.352	0.406	0.493	0.520	0.578	0.612
5	0.411	0.465	0.545	0.584	0.658	0.701
10	0.481	0.527	0.601	0.642	0.704	0.726
15	0.594	0.627	0.683	0.700	0.743	0.760
20	0.699	0.719	0.757	0.775	0.794	0.799
25	0.784	0.792	0.812	0.825	0.852	0.849
30	0.848	0.846	0.862	0.862	0.869	0.879
35	0.907	0.891	0.897	0.900	0.898	0.888
40	0.948	0.936	0.932	0.916	0.937	0.969
45	0.978	0.963	0.955	0.942	0.952	0.943
50	1.002	0.986	0.974	0.961	0.963	0.971
55	1.017	1.012	0.997	0.980	0.979	0.954
60	1.029	1.024	1.008	0.993	0.990	1.001
65	1.033	1.030	1.024	1.009	0.994	0.998
70	1.029	1.039	1.027	1.006	1.016	1.010
75	0.997	1.043	1.031	1.018	1.021	0.999
80	0.999	1.025	1.024	1.023	1.009	1.025
85	0.999	0.999	1.009	1.006	0.994	1.007
90	1.000	1.000	1.000	1.000	1.000	1.000

$$\phi_{an}(r)=\frac{\int_{o}^{\pi }\dot{D}(r,\theta )\sin\;\theta d\theta }{2\dot{D}(r,\theta_{o})}.$$

The anisotropy constant, ${\bar{\phi }}_{\text{an}}$, was calculated by averaging the anisotropy factors over all radii. Values in the present study range from 0.941 and 0.944 for the BT‐125‐1 and BT‐125‐2 sources to 0.960 for the ADVANTAGE source. Good agreement was observed between TLD measurements and Monte Carlo calculations in the present study. Additionally, values are consistent with reports on two other commercially available sources (Table VIII).^39^ ^,^ ^40^ ^,^ ^42^

**Table VIII:** Anisotropy factors determined in the present work compared with that of previous studies in which solid core, cylindrical sources were evaluated.

Reference	Technique of determination	Medium	Anisotropy constant	${\bar{\phi }}_{\text{an}}$
Present work BT‐125‐1 (Pd‐core)	Monte Carlo	Water	0.941
Popescu *et al*. BT‐125‐1 (Pd‐core)	Monte Carlo	Water	0.974
Popescu *et al*. BT‐125‐1 (Pd‐core)	TLD Measurements	Solid Water™	0.969
Present work BT‐125‐2 (Ag‐core)	Monte Carlo	Water	0.944
Present Work Advantage	Monte Carlo	Water	0.960
Present Work Advantage	TLD Measurements	Plastic Water™	0.950
Kirov and Williamson STM1251	Monte Carlo	Water	0.941
Weaver Model 6711	Monte Carlo	Water	0.948

## DISCUSSION

Monte Carlo methods have found increasing application in a wide variety of medical physics and radiation dosimetry problems. The concept of applying Monte Carlo techniques to brachytherapy

originated in the early 1980s.^43^ ^,^ ^45^ Since that time there has been extensive research in this area. Among the foremost proponents of the technique have been Williamson and colleagues. Using a Monte Carlo code of their own design, incorporating photon transport only (MCPT), Williamson calculated some of the first dosimetric parameters for low energy sources and pointed out significant discrepancies with then‐existing data.^17^ ^,^ ^18^ Much of this original data calculated by Williamson has found its way into commercial treatment planning systems.

While MCNP is less commonly used in general medical physics applications than other Monte Carlo codes, EGS4 in particular, the use of MCNP in dosimetry of low and medium energy β emitters and γ emitters is nevertheless well documented. In the early 1990s, Mason *et al*. and MacPherson and Battista used MCNP to calculate dosimetric parameters of Yb169, a new low energy brachytherapy source.^46^ ^,^ ^47^ The authors used MCNP to accurately model the seeds to account for factors such as photon attenuation, self‐absorption, and scattering. As in the present work, dose was determined by scoring the energy fluence (using a track length estimator) and multiplying by the mass‐energy absorption coefficient. Calculated dose rate constant and angular dose profiles agreed with TLD measurement within approximately 5%, particularly good considering the now obsolete low energy photon cross sections used in MCNP4A and recently pointed out by other investigators.^48^ Calculated source strength agreed with measurement within the statistical uncertainty of both techniques.

**Table VI B:** Coefficients for anisotropy function fit to a fifth order polynomial.

Fitting coefficients for F(r,θ) Distance from source center (cm)						
$a_{0}$	3.46E–46	4.01E–01	4.89E–89	5.20E–20	5.83E–83	6.25E–25
$a_{1}$	1.07E–07	1.07E–07	9.88E–88	1.16E–16	1.34E–34	1.07E–07
$a_{2}$	6.59E–59	5.17E–17	3.81E–81	1.92E–92	–1.31E–31	–5.25E–25
$a_{3}$	–2.11E–11	–1.84E–84	–1.47E–47	–1.02E–02	5.19E–19	–1.26E–26
$a_{4}$	2.18E–18	2.13E–13	1.78E–78	1.36E–36	9.01E–01	2.00E–00
$a_{5}$	–7.72E–72	–8.68E–68	–7.58E–58	–6.07E–07	–6.04E–04	–9.41E–41

**Table VII:** Anisotropy factors determined in the present work compared with that of previous studies in which solid core, cylindrical sources were evaluated.

Anisotropy factors, $\phi_{\text{an}}(r)$							
		Present work			Popescu *et al*.	Kirov and Williamson	Weaver
Radial distance (cm)	BT‐125‐1 Monte Carlo	BT‐125‐2 Monte Carlo	Advantage Monte Carlo	Advantage TLD	BT‐125‐1 Monte Carlo	STM1251 Monte Carlo	6711 Monte Carlo
0.5	0.933	0.931	0.957	1.000	1.000		0.973
1.0	0.948	0.946	0.968	0.960	0.976	0.942	0.944
2.0	0.946	0.946	0.964	0.930	0.973	0.937	0.941
3.0	0.943	0.943	0.955	0.940	0.970	0.947	0.942
4.0				0.950		0.941	0.943
5.0	0.940	0.948	0.959	0.950	0.968	0.938	0.944
6.0				0.940			
7.0	0.937	0.949	0.955		0.964	0.944	

Wierzbicki *et al*. used MCNP to calculate dosimetric parameters of a commercial I125 source following the AAPM Task Group 43 recommendations.^49^ Radial dose function, anisotropy factors, and anisotropy constants were determined by scoring the absorbed dose (using the MCNP *f8 tally) in a spherical water phantom. The seeds, consisting of four I125‐impregnated resin beads encapsulated in Titanium tubes, were modeled in their geometric entirety. Small differences between calculated values and those measured by previous investigators were observed. The authors speculated that the differences were primarily due to the phantom materials used in earlier measurements and recommend the use of the Monte Carlo values for clinical applications.

Subsequently, Rivard used MCNP to investigate the validity of point and line source approximations in the determination of geometry factors.^41^ The author used a different geometry scheme from the present work, requiring the simulation of a much larger number of incident particles, but nonetheless concluded there was significant merit in using Monte Carlo to calculate geometry factors as compared with standard approximations. Use of this methodology will take on even greater importance with newer sources that may deviate significantly from a pure line source.

Most recently DeMarco *et al*. used MCNP to model two‐ and three‐dimensional dose distributions from permanent I125 implants for carcinoma of the prostate.^48^ The authors took advantage of the voxel‐based geometry feature of MCNP to investigate the effect of tissue heterogeneities on the resulting CT‐based dose distribution resulting from multiple seed configurations.

Finally, Wuu *et al*. used MCNP to generate the electron slowing down spectrum for a second code, delta, to estimate the relative biological effectiveness (RBE) of four low‐medium energy sources relative to C60o.^50^ Various groups have used MCNP in medium energy (^192^Ir) brachytherapy dosimetry^51^ ^–^ ^54^ as well as in intravascular brachytherapy applications.^55^ ^–^ ^57^ And while significantly different from the present work, several groups have used MCNP to assist with dosimetric calculations for neutron‐emitting brachytherapy sources.^58^ ^,^ ^59^

In this work we have used MCNP to calculate dosimetric parameters of three I125 sources per the AAPM Task Group 43 protocol. Results compare favorably with our own TLD measurements and with Monte Carlo calculations of a prior study (evaluating the Syncor source) by Popescu *et al*.^35^ Additionally, our results are compared with two other commercially available I125 sources of similar geometric construction. In particular, dosimetric characteristics of both the Syncor and IsoAid sources, including dose rate constant and two‐dimensional dose distribution, are essentially indistinguishable from the widely used model 6711. Differences were observed, however, when compared to the Source Tech Medical model STM1251. The authors attribute this to a longer source length, a low density (gold) core, and a thin Ni and Cu coating.^40^ Multi‐element construction of this type can present significant challenges for the Monte Carlo methodology in the production and transport of the resulting low energy phenomena.

In this work, the radial dose and anisotropy functions derived from Monte Carlo techniques were each normalized by a geometry function that was also obtained through Monte Carlo calculations. While the sources studied were simple and regular in design, differences in the geometry function from the standard line source approximation were nevertheless observed close to the source. Designs that vary considerably from the solid cylindrical core construction will exhibit even larger differences.

In summary, MCNP is an excellent tool for determining dosimetric parameters in any medium, most importantly, in water. The radiation transport capabilities of MCNP, particularly at the lower energies applicable to permanent seed brachytherapy, have been thoroughly studied and are well documented throughout the literature. Source designs can be visualized within the application, simplifying problem construction and providing added assurance that the source and phantom geometries have been modeled correctly and in appropriate detail. MCNP is capable of deriving an exact geometry function for any source configuration. These capabilities will take on increasing significance as new sources with more sophisticated geometries become available. Thus, MCNP appears to be ideally suited to low energy dosimetry applications and will undoubtedly find increasing use in this area.
